# Of first impressions, shattered trust, and apology: impact on interpersonal trust and team dynamics

**DOI:** 10.3389/fpsyg.2025.1654463

**Published:** 2025-08-07

**Authors:** Majlinda Maliqi, Fanny Lalot, Alain Quiamzade

**Affiliations:** ^1^Faculty of Psychology, University of Basel, Basel, Switzerland; ^2^Faculty of Psychology and Educational Sciences, Universite de Geneve, Geneva, Switzerland; ^3^FernUni Schweiz Fakultat Psychologie, Brig, Switzerland

**Keywords:** congruence bias, belief updating, first impressions, trust, trustworthiness

## Abstract

**Introduction:**

This study aimed to investigate the dynamics of trust formation in the work context. Specifically, the study aims to test how first impressions and new information about a new team member (1) interact to determine interpersonal trust in this person, and (2) influence perceptions of the wider work team.

**Methods:**

We present the findings of a preregistered experimental study conducted amongst employees in Northwestern Switzerland (*N* = 204). We relied on a hiring paradigm, using a bogus job interview video to manipulate first impressions of a job candidate through her response to an accusation of past trust violation (denial vs. apology). This was followed by new positive information about the job candidate. Outcomes included the perceived trustworthiness of, and trust in the job candidate, as well as the anticipated team dynamics if the person were to join the participant’s work team.

**Results:**

Contradicting a congruence bias hypothesis, the results showed a sustained positive effect of first impressions (specifically, the impact of apology over denial), demonstrating an additive, rather than multiplicative, positive effect of the new information on (1) perceived trustworthiness and interpersonal trust as well as (2) collaborative culture, perceived performance, and satisfaction with team functioning.

**Discussion:**

We highlight theoretical implications for belief updating research and suggest applications for trust interventions in the workplace.

## Introduction

The contemporary working environment is characterised by a team-based structure: across domains and organisations (including academia), more people work in teams than alone (e.g., Kozlowski and Ilgen, 2006; Moe et al., 2009; Osterman, 1994; Wuchty et al., 2007). Furthermore, the working environment has undergone significant changes in recent years, including the widespread adoption of remote work and hybrid offices, which have led to the creation of more virtual teams and increased reliance on electronic communication tools (e.g., Kulykovets, 2024). In such settings, effective team dynamics become crucial to both task performance and job satisfaction. One vital element for harmonious teamwork is *trust* (Breuer et al., 2016; Costa et al., 2018; McAllister, 1995). Yet, trust is fragile and, once damaged, difficult to repair (Lewicki and Brinsfield, 2017). Specifically, doubts about the trustworthiness of just one coworker may challenge the dynamics of the entire team. However, the extent to which interpersonal trust dynamics may influence team perceptions remains underexplored.

The aim of this paper is twofold. At the theoretical level, we shed light on the dynamics of trust formation in the work context, specifically examining the interplay between first impressions and new information in determining interpersonal trust. Second, at the applied level, we aim to investigate how individual perceptions of a team may be challenged by the anticipated arrival of a new team member, especially when the trustworthiness of that member is in question.

This paper presents the findings of a preregistered experimental study conducted amongst employees in Northwestern Switzerland. We relied on a hiring paradigm and presented participants with a video clip of a bogus job interview, asking them to evaluate the job candidate. This paradigm was used to manipulate the candidate’s response to an accusation of past trust violation (denial vs. apology) and served as a test of “first impression.” We then provided participants with a piece of positive information about the candidate to assess how they processed new information in relation to their first (positive or negative) impression. We finally measured perceived trustworthiness and trust in the candidate, as well as anticipated team dynamics, if this person were to join the participant’s work team. Our results show a sustained positive effect of apology over denial, with an additive, but not multiplicative, positive effect of the new information on perceived trustworthiness and trust, as well as subjective perceptions of collaborative culture, team performance, continuance commitment, and satisfaction with team functioning. In the following sections, we develop our theoretical reasoning for the study and state our preregistered hypotheses before turning to the description of the study.

### Defining trust

Here, we adopt a widely accepted definition of *trust* put forth by Rousseau et al. (1998, p. 395): “a psychological state comprising the intention to accept vulnerability based upon positive expectations of the intentions or behaviour of another.” Trust is thus conceived as an attitude that will be weighed against perceived risk to determine risk-taking in a given situation. As such, it is conceptually distinct from (albeit an important predictor of) trusting behaviour, which we refer to as *willingness to risk* (see Kim et al., 2004).

Trust must also be distinguished from trustworthiness, which reflects the characteristics of the trustee. *Trustworthiness* includes at least three elements: the trustee’s ability (“that group of skills, competencies, and characteristics that enable a party to influence within some specific domain,” Mayer et al., 1995, p. 717), benevolence (“the extent to which the trustee is believed to want to do good to the trustor, aside from an egocentric profit motive,” ibid., p. 718), and integrity (“the perception that the trustee adheres to a set of principles that the trustor finds acceptable,” ibid., p. 719; see also Colquitt et al., 2007; Kelton et al., 2008; Schoorman et al., 2007). Trustworthiness is evaluated through third-party information and observation of the trustee’s actions, and gradually develops as more information becomes available (Hung et al., 2004; Mayer et al., 1995).

Others have distinguished between *cognitive trust* and *affective trust* (McAllister, 1995). Cognition-based trust is rooted in searching evidence or clues of trustworthiness, building upon “good reasons” to trust the person [primarily based on the ability and integrity components of trustworthiness (Tomlinson et al., 2020)]. Affect-based trust, on the other hand, is built on the emotional bonds between individuals, which grow stronger as the relationship develops (McAllister, 1995; Tomlinson et al., 2004) and corresponds mostly to perceived benevolence and shared values (Tomlinson et al., 2020).

### Trust building: from first impressions to beliefs updating

People quickly form first impressions of others’ trustworthiness, before even meeting them. These first impressions are built based on various cues such as facial features (Todorov et al., 2008), social indicators like in-group membership (Hughes et al., 2017), and third-party information (Ferrin et al., 2006). This initial impression is then updated as people interact and gather new information about the trustee’s trustworthiness (Hung et al., 2004; Mayer et al., 1995). As the relationship consolidates, trust judgments become more resistant to new information (Cvetkovich et al., 2002; Dietz and Den Hartog, 2006). They thus evolve from a cognitive base to a more affective one (McAllister, 1995).

Different models suggest ways in which trust beliefs are updated in response to new information. First, the asymmetry principle states that negative information has a greater impact than positive information (Slovic, 1993; see also Kahneman and Tversky, 1979). Therefore, and not surprisingly, trust is more easily lost than built (Lewicki and Brinsfield, 2017). Furthermore, information is not always given equal weight. Research has highlighted a belief-congruent or confirmatory bias, wherein new information that is congruent with initial beliefs is given more consideration (Cvetkovich et al., 2002). For example, Poortinga and Pidgeon (2004) found that participants’ prior beliefs moderated the importance they assigned to new positive vs. negative information about genetically modified food. While early research mostly focused on risk assessment (e.g., Cvetkovich et al., 2002; Poortinga and Pidgeon, 2004; Slovic, 1993), other evidence suggests that judgments of individuals’ trustworthiness are similarly biased, be it as a function of the trustee’s reputation (Pilditch et al., 2020) or implicit initial trustworthiness information (Chang et al., 2010). Experimental research consistently suggests that early trust violations make it very difficult to restore trust even when the trustee later demonstrates consistently trustworthy behaviour (Lount et al., 2008). In summary, it appears that trustors do not simply take in and summarise new information, updating their trust beliefs as they go. Instead, they treat this information in a way that is biased both cognitively (e.g., the asymmetry bias) and motivationally (e.g., the belief-congruent bias).

### Trust in the workplace

What about trust perceptions in the workplace? Organisational trust can be apprehended at many levels and in different forms, from the inter-organisational level (i.e., between organisations) to the intra-organisational level (encompassing trust in different referents, such as the organisation itself, one’s manager and coworkers, etc., see Dietz and Den Hartog, 2006; Fulmer and Gelfand, 2012; Tyler, 2003). Here, we focus on the processes of team trust and specifically on interpersonal trust among team members.

*Team trust* represents the aggregated perception of trust in the team as a distinct unit or in its members collectively (Costa, 2003; Costa et al., 2018). Different theories suggest that team trust stems from interpersonal trust between team members (Costa et al., 2018; Kozlowski and Klein, 2000; Marks et al., 2001). Recent work also demonstrated that initial trust judgments emerge quickly, even in short-term teams, and that such individually held perceptions influence the construction of team trustworthiness over time (Fletcher et al., 2024).

While team trust may be a desirable feature in itself, its importance stems mostly from its vital role in fostering harmonious teamwork (Costa et al., 2018; McAllister, 1995). Among other things, trust has been found to contribute to increased perceptions of collaborative culture (Barczak et al., 2010) and team performance (Costa et al., 2001; Erdem et al., 2003; see also Grossman and Feitosa, 2018), as well as greater team satisfaction (Braun et al., 2013; Chou et al., 2008) and continuance commitment (Costa et al., 2001; Matzler and Renzl, 2006; for reviews, see, e.g., Breuer et al., 2016; Costa et al., 2018; De Jong et al., 2016).

### The present research: from coworker trust building to team perceptions

Previous research on belief updating has explored interpersonal trust in general, but has rarely addressed dynamics specific to the workplace (for exceptions, see, e.g., Campagna et al., 2022; van der Werff and Buckley, 2014). In the present study, we aimed to extend research on trust in the workplace by integrating findings on trust based on first impressions. We thus investigate building trust *in a new coworker* and its downstream consequences. Going beyond just interpersonal dynamics, we consider how the dynamics of building and updating trust beliefs about this coworker might then translate into different perceptions of one’s work team (as listed above), in anticipation of the coworker joining it. Indeed, previous studies have shown that the arrival of a new coworker may shake up existing team dynamics (Allen and Meyer, 1990; Bauer et al., 2007; Liu et al., 2023) and threaten trust amongst team members. Thus, this study aims to provide a thorough assessment of trust formation and belief updating in the workplace by articulating theoretical frameworks that, to date, have remained largely disconnected.

In the present study, we utilised an experimental design that allowed us to isolate the specific mechanisms of interest, all else being equal (e.g., potential confounds related to participants’ diverse experiences in their workplace). We manipulated the first impression of an alleged future coworker as more or less trustworthy before introducing new positive information about the person. This allows us to test how initial trust beliefs are updated. We then turned to team-related beliefs, assessing participants’ beliefs about how team dynamics may be affected if and when the new coworker joined their team.

A strength of this research is that our sample is composed of employees (rather than just university students), ensuring an understanding of workplace dynamics and allowing participants to project themselves into a potential future where the job candidate would join their real-life work team. This extends previous findings that only considered graduate and undergraduate students. We now outline the various components of the study, along with the related preregistered hypotheses.

#### First impression: denial and apology

We adapted a bogus job interview paradigm (Kim et al., 2004, 2006) to introduce a past work violation related to a lack of competence, which is disclosed during the interview, and to manipulate the job candidate’s response in terms of either denial or apology for the violation. Kim and colleagues previously used this paradigm to assess how disclosure of a past trust violation affected participants’ evaluation of a job candidate, and how different reactions from the job candidate moderated this evaluation. They found that acknowledging and apologising for an ability-related violation was more effective in terms of restoring trust and hiring intentions (whereas denial was more effective for an integrity-related trust violation; Ferrin et al., 2007; Kim et al., 2004, 2006).

We decided to focus on situations of competence-based trust violation given their widespread occurrence in the workplace and strong impact on trust, especially employee trust (Kähkönen et al., 2021). We manipulated the candidate’s response to induce more positive or negative first impression (rather than, for example, comparing conditions of violation vs. no violation) to provide a more nuanced perspective on the formation and updating of trust beliefs. Based on Kim et al. research (Kim et al., 2004, 2006), we hypothesised that:

*H1*: Participants will report higher trust (measured at T1) towards the job candidate (and describe them as more trustworthy) when the person apologises than when they deny the competence-based trust violation. Note that this first hypothesis reflects a direct replication of a well-established effect, and we merely consider it as such.

We then introduced the new positive information about the job candidate (see Methods below). In line with confirmation bias research (e.g., Cvetkovich et al., 2002; Pilditch et al., 2020; Poortinga and Pidgeon, 2004), we expected that participants would treat this new information in a way that is congruent with their first impression based on the candidate’s behaviour during the interview (i.e., denial or apology). Specifically, we hypothesised that:

*H2*: After having received additional positive information about the job candidate (T2), participants will report a greater *increase* in trust when the candidate initially apologised than when they denied the violation.

We then turned to team-related perceptions, thus extending first-impressions research beyond interpersonal trust. We asked participants to imagine that the job candidate was eventually hired and would soon join their team. Based on the notion that team trust stems from interpersonal trust between team members (Costa et al., 2018; Kozlowski and Klein, 2000; Marks et al., 2001) and is influenced by changes in the team’s composition, we assessed differences in perceived collaborative culture (Barczak et al., 2010), anticipated team performance (Erdem et al., 2003), satisfaction with team functioning (Braun et al., 2013), and continuance commitment (Costa et al., 2001) – all variables known to be influenced by team trust. We expected the initial manipulation (response to the trust violation) would influence each of the above. Specifically, participants in the Apology condition, compared to those in the Denial condition, should report:

*H3a*: …higher perceived collaborative culture within their team.

*H4a*: …higher anticipated team performance.

*H5a*: …higher continuance commitment within their team.

*H6a*: …higher satisfaction within their team.

In addition, assuming that this effect reflects a change in the perceived trust of the job candidate, we expected trust to mediate the effects of the experimental manipulation. We thus hypothesised that trust (measured at T2, that is, after the additional positive information was disclosed) would mediate the effect of the experimental manipulation on:

*H3b*: …perceived collaborative culture.

*H4b*: … anticipated team performance.

*H5b*: …continuance commitment.

*H6b*: …satisfaction with team functioning.

Finally, based on findings that satisfaction with team functioning also derives from perceived performance (Costa et al., 2001), we hypothesised the following serial mediation:

*H6c*: The effect of the experimental manipulation on satisfaction with team functioning is serially mediated through trust and then anticipated team performance.

### Research transparency statement

The study design, hypotheses, materials, sample size, and exclusion criteria were preregistered: https://aspredicted.org/sfkb-sg4t.pdf. Data, materials, and code for analysis are publicly available on the OSF: https://osf.io/vyzkx. This research was approved by the ethics committee of the Faculty of Psychology at the University of Basel, and all participants gave informed consent prior to their inclusion in the study.

## Methods

### Participants and procedure

We invited employees from Northwestern Switzerland to participate in an online study. Conditions for participation were being employed in a team of at least three members and being fluent in German. A partnering company agreed to distribute the study directly to their employees. We also advertised it on social media and through the university network. Participants completed the study voluntarily and were eligible to enter a draw to win one of several vouchers as a token of appreciation. As indicated in the preregistration, we determined the sample size based on an *a priori* power analysis, aiming to detect small-to-medium effect sizes (i.e., Cohen’s *d* = 0.40). The analysis indicated that a sample of *N* = 200 would allow us to detect such an effect with 0.80 power (alpha = 0.05), so we set our target sample size at 200.

Two hundred and fourteen participants completed the survey. As preregistered, we excluded participants who failed an attention check embedded in the survey (*n* = 3), those who worked in teams smaller than three members (*n* = 3), and those who were not currently working (*n* = 4). The final sample consisted of *N* = 204 (83 men, 118 women, one non-binary individual, and two did not disclose their gender; *M*_age_ = 33.97, *SD* = 12.27).^1^

**Figure 1:**
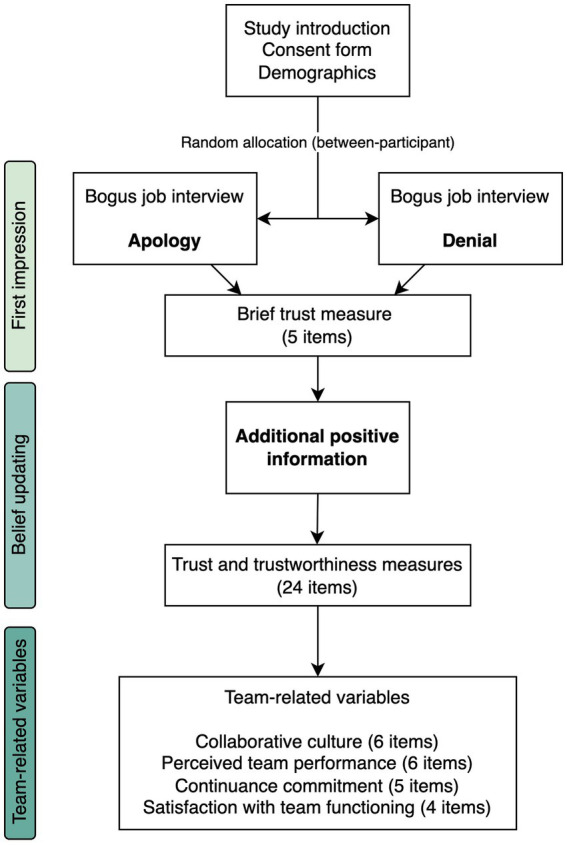
Study procedure.

The study was introduced as an investigation of team dynamics in the work context. Figure 1 depicts the study procedure. We asked participants to imagine that their work team was in the process of recruiting a new member, about whom they would now receive some information. They first watched a short video of a bogus job interview, which served as our experimental manipulation of the first impression of the (female) job candidate based on her response (see below). They were randomly allocated to either the *Apology* (*n* = 104) or *Denial* (*n* = 100) condition. They completed a brief measure of trust perception before being presented with new information about the candidate. This positive information was presented to all participants. Then, they completed a longer set of items that measured cognitive and affective trust in the candidate, perceived trustworthiness, and willingness to risk. Following this, we asked them to imagine that the job candidate had been hired and would now join their team, and to reflect on how this might affect team dynamics. They then completed the final measures of collaborative culture, perceived team performance, continuance commitment, and satisfaction with team functioning. They were finally thanked, debriefed, and allowed to enter a lottery to win a voucher.

### Materials

#### First impression manipulation

We designed and filmed a bogus job interview to manipulate the first impression of the job candidate. The video focused on the pretended job candidate (a female actor); the interviewer could be heard speaking but was not visible in the video frame. The excerpt started with a neutral conversation, where the candidate introduced herself as Ms. Anna Bloom and described her motivation for applying for this job. The job details were intentionally kept vague so that all participants could relate to the content regardless of their professional role. The experimental manipulation was then introduced as the interviewer revealed that the candidate’s previous employer, who had been contacted to provide a reference, disclosed a significant fault related to a competence-based trust violation during her employment. Specifically, the candidate was accused of forwarding an email with sensitive information to the entire department rather than to her supervisor. Her response served as the experimental manipulation.

In the *Denial* condition, she denied the accusation and blamed unclear instructions she had received from her supervisor. Conversely, in the *Apology* condition, she acknowledged the unintended mistake, apologised, and explained it was due to her unfamiliarity with creating a distribution list. In both conditions, she concluded by reassuring the interviewer that such an incident would never occur again if she were hired. The video stopped there.

A few questions followed the video and were intended to verify that the denial response led to lower trust and perceived trustworthiness than the apology (i.e., direct replication of Kim et al., 2004, 2006). Out of concern for keeping the study as brief as possible, we only included five items, measuring the job candidate’s perceived ability (“I have confidence in Anna’s abilities”), benevolence (“Anna has good intentions”), integrity (“Anna is honest and open”), as well as willingness to risk (“I would entrust Anna with a task or problem that is important to me, even if I could not monitor her actions,” 1 = *Strongly disagree*, 7 = *Strongly agree*), and general trust (“How much do you trust Anna?,” 1 = *Not at all*, 7 = *Very much*).

#### Additional positive information

We then provided all participants with new information about the job candidate, in the form of a fictitious animated chat conversation (generated using the TypeStory app for iOS) between the interviewer and a colleague. Our goal was to provide positive, albeit non-work-related, information about the candidate. The interviewer shared a link to an excerpt from a local newspaper, which mentioned the job candidate and the role she played in maintaining a local volleyball club afloat through her dedication and long-term commitment to it. A brief conversation ensued about the content of the article. This information was maintained constant across the two experimental conditions.

#### Dependent measures (1): trust and trustworthiness following additional information

Following the additional positive information, we asked participants to evaluate the job candidate on several dimensions. All items used a 7-point Likert scale. The questionnaire included measures of perceived *trustworthiness* as a combination of ability (four items, e.g., “I have confidence in Anna’s abilities”), benevolence (five items, e.g., “Anna has good intentions”), and integrity (four items, e.g., “Anna is honest and open,” Lee et al., 2022); of *cognitive* (four items, e.g., “Anna does her job with professionalism and dedication”) and *affective trust* (three items, e.g., “I could talk openly with Anna about my ideas, feelings, and hopes,” McAllister, 1995), *willingness to risk* (three items, e.g., “I would entrust Anna with a task or problem that is important to me, even if I could not monitor her actions,” Ferrin et al., 2007), and a self-developed single item measuring *general trust* in the candidate (“How much do you trust Anna?”). The measures therefore repeated the items used as a direct replication of Kim and colleagues (directly after the video), allowing for direct pre-post change tests, while also aiming to increase measurement reliability by including a larger number of items.

#### Dependent measures (2): team-related variables

Participants then completed a few measures assessing team dynamics, assuming the job candidate would now join their own team as a coworker. Specifically, we measured perceptions of *collaborative culture* (six items, e.g., “Collaboration and co-operation between team members will be encouraged,” Barczak et al., 2010), *perceived team performance* (six items, e.g., “With Anna in our team, we will be known to perform better than other teams,” Horn et al., 2011), *continuance commitment* (five items, e.g., “I will think about changing my team or finding work elsewhere once Anna is part of the team,” reverse-coded, Horn et al., 2011), and *satisfaction with team functioning* (four items, e.g., “Overall, I will be quite satisfied with our working relationship once Anna is here,” Smith and Barclay, 1997).

## Results

The code for analysis is available in Electronic Supplementary Materials (ESM1) on the OSF: https://osf.io/vyzkx. As preregistered, we first conducted a factor analysis to determine how to handle the measurement of trust-related constructs in the best way. The full output of these analyses is reported in ESM2 (Supplementary material).

### Apology vs. denial: test of direct replication

#### Exploratory factor analysis

We conducted an exploratory factor analysis on the five items serving as a direct replication of Kim et al. (2004, 2006). We relied on a multiple-criteria approach to determine the number of factors to extract (Ruscio and Roche, 2012). There was a consensus among the comparison data index, parallel analysis, sequential χ^2^ model tests, and the lower bound of 90% CI of RMSEA, which all recommended extracting two factors/dimensions. We therefore conducted an exploratory factor analysis (EFA) with extraction fixed on two factors (maximum likelihood). The solution (explaining 77% of variance) revealed a first factor grouping the three items of trustworthiness and that of general trust (61% variance, all loadings > 0.48) and a second factor on which the willingness-to-risk item loaded alone (16% variance, loading = 0.99). Given their psychometric unidimensionality, we therefore aggregated the first four items into an index of trust/trustworthiness despite their theoretical distinction. While the trust literature has highlighted conceptual distinctions between trustworthiness and trust, this is not always reflected at the measurement level, especially when relying on a limited number of items. For simplicity’s sake, we thus followed the data structure. It is worth noting that similar results were obtained when considering trust and trustworthiness separately (see below). We maintained willingness to risk as a separate, single-item indicator reflecting the second factor.

#### Analysis of variance (ANOVA)

We conducted two ANOVAs to test whether the manipulation of trustworthiness (i.e., the job candidate responding by denying vs. apologising for the trust violation) impacted trust as expected. The first analysis showed a significant effect on perceived trust/trustworthiness: the job candidate was rated as more trustworthy and trusted when she apologised (*M* = 5.34, *SD* = 1.16) than when she denied the violation (*M* = 4.58, *SD* = 1.14), *F*(1, 202) = 22.54, *p* < 0.001, Cohen’s *d* = 0.67, 95% CI [0.38, 0.94].^2^

Similarly, participants reported higher willingness to risk towards the candidate when she apologised (*M* = 5.02, *SD* = 1.49) than when she denied the violation (*M* = 3.98, *SD* = 1.60), *F*(1, 202) = 23.08, *p* < 0.001, *d* = 0.67, 95% CI [0.39, 0.95]. This directly replicated the pattern of results observed by the authors who originally developed this paradigm (H1).

### Trust and trustworthiness following additional information

#### Confirmatory factor analysis and measurement model

Given the greater number of items used for trust ratings after additional positive information about the job candidate was revealed, we relied on a confirmatory factor analysis instead of an EFA. We specified a model in which each theoretical construct was composed of several items (see list above), except for general trust, which consisted of a single item. Furthermore, we specified a higher-order construct of trustworthiness, composed of three latent constructs of ability, benevolence, and integrity. While this model yielded acceptable model fit (χ^2^ = 581.38, df = 240, χ^2^/df = 2.42, CFI = 0.912, RMSEA = 0.084, 90% CI [0.075, 0.092], SRMR = 0.062), results revealed some items that did not load significantly or strongly on their theoretical construct. This led us to remove four underperforming items (one ability, two benevolence, one cognitive trust), which improved the model fit [χ^2^ = 388.74, df = 158, χ^2^/df = 2.46, CFI = 0.938, RMSEA = 0.085, 90% CI [0.074, 0.095], SRMR = 0.050; see details in ESM2 (Supplementary material)]. All descriptive statistics and reliability indices for multi-item measures are reported in Table 1.

**Table 1:** Descriptive statistics and reliability indices for all outcome measures.

#	Construct	Descriptive statistics		Pearson’s correlations								
		Cronbach’s α	*M* (*SD*)	2	3	4	5	6	7	8	9	10
1	Trustworthiness	0.93	4.98 (0.82)	0.78^***^	0.83^***^	0.67^***^	0.70^***^	0.96^***^	0.37^***^	0.71^***^	0.37^***^	0.73^***^
2	General trust	-	4.86 (1.23)		0.79^***^	0.67^***^	0.85^***^	0.83^***^	0.28^***^	0.69^***^	0.41^***^	0.67^***^
3	Cognitive trust	0.88	4.96 (1.19)			0.63^***^	0.76^***^	0.89^***^	0.26^***^	0.74^***^	0.44^***^	0.66^***^
4	Affective trust	0.89	4.66 (1.24)				0.58^***^	0.81^***^	0.34^***^	0.61^***^	0.40^***^	0.63^***^
5	Willingness to risk	0.87	4.86 (1.36)					0.71^***^	0.21^**^	0.70^***^	0.40^***^	0.60^***^
6	Aggregate index of trust	0.96	4.70 (0.71)						0.38^***^	0.75^***^	0.42^***^	0.77^***^
7	Collaborative culture	0.84	5.10 (0.99)							0.37^***^	0.41^***^	0.52^***^
8	Team performance	0.85	5.08 (0.96)								0.47^***^	0.77^***^
9	Continuance commitment	0.73	5.67 (0.98)									0.59^***^
10	Satisfaction with team	0.88	4.90 (1.10)									

All items are measured on a 7-point Likert scale. ^**^ *p* < 0.01, ^***^ *p* < 0.001.

#### ANOVA

We then tested the effect of the first impression manipulation on trust ratings *after* participants were also presented with additional positive information about the job candidate (i.e., belief updating). As per our preregistered hypothesis, we expected participants to process this new information in a congruence-bias-consistent manner, so that participants in the Apology condition would polarise their judgements towards even greater positivity, while participants in the Denial condition would resist this new incongruent piece of information and change their judgements less (H2).

##### Pre-post change

We first examined the five items that were repeated verbatim before and after the additional positive information (i.e., as a direct replication test). Contrary to expectations, the analysis revealed no significant effect: the change in trust/trustworthiness ratings was not significantly different in the Apology (*M* = 0.12, *SD* = 0.70) and the Denial condition (*M* = 0.68, *SD* = 0.31), *F*(1, 202) = 3.80, *p* = 0.053, *d* = 0.27, 95% CI [−0.003, 0.54]. If anything, any trending effect was going in the opposite direction to the prediction, with marginally greater change in the Denial condition. The pre-post change was significantly different from zero, indicating an overall improvement in ratings, *t*(203) = 4.43, *p* < 0.001.^3^

Turning to willingness to risk, change in means was not different across conditions (*M*_Apology_ = −0.10, *SD* = 1.27, *M*_Denial_ = 0.14, *SD* = 1.33), *F*(1, 202) = 1.69, *p* = 0.20, *d* = 0.18, 95% CI [−0.09, 0.45]. Scores did not change significantly from pre- to post-test overall, *t*(203) = 0.22, *p* = 0.83. Our hypothesis was thus not supported: the new information did not seem to be processed in a congruence-bias-consistent way. Results suggest instead a linear and additive effect of this new information, which ameliorated trust ratings (but not willingness to risk) to the same extent in both experimental conditions.

##### Trust ratings following the new positive information

**Figure 2:**
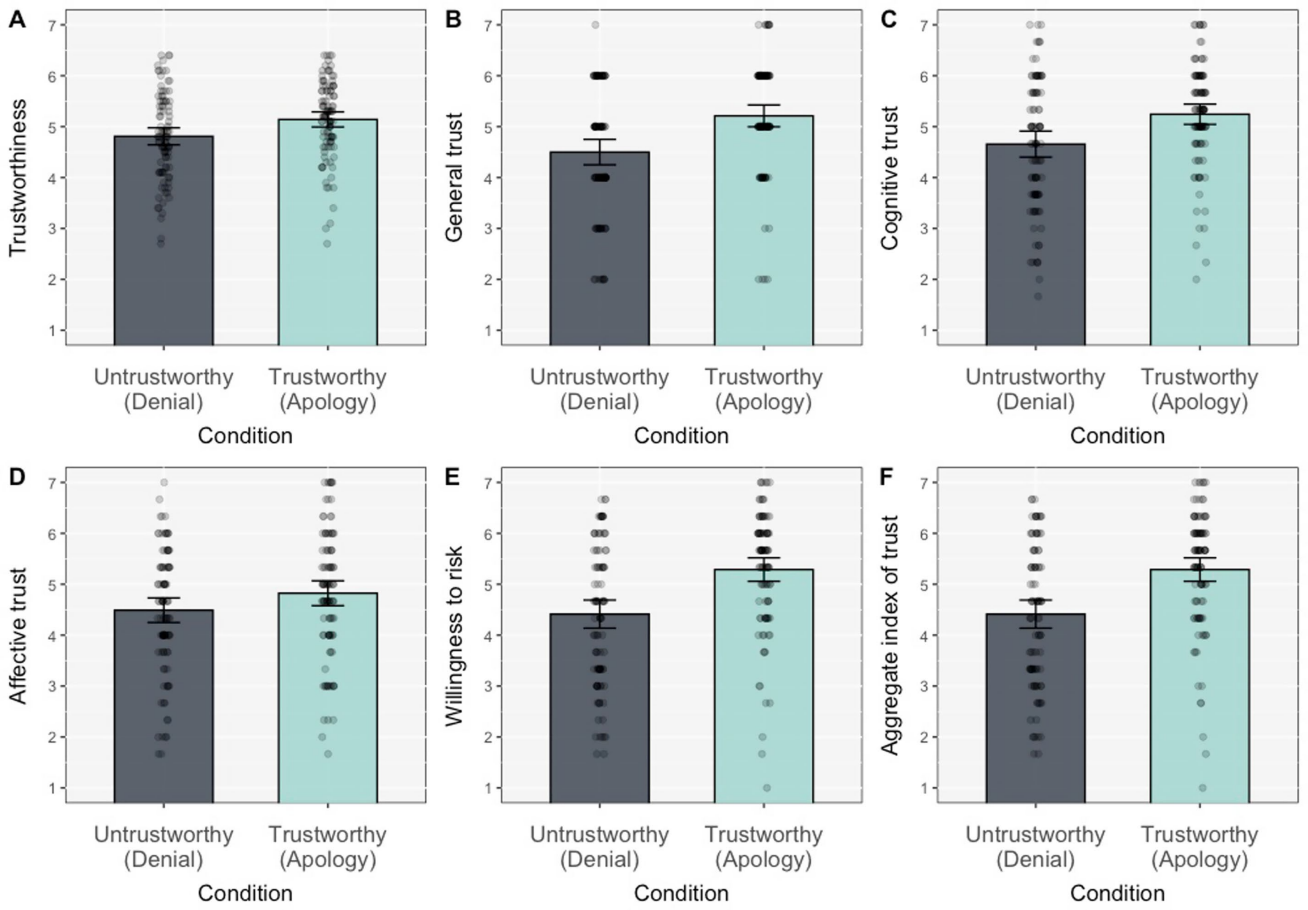
Effect of the first impression manipulation (condition: denial vs. apology) on ratings of trustworthiness **(A)**, general trust **(B)**, cognitive trust **(C)**, affective trust **(D)**, willingness to risk **(E)**, and trust as an aggregate index **(F)**, following the new positive information. All differences were significant at *p* < 0.01 except for affective trust **(D)** where it was not significant.

In a second step, we turned to the full set of items assessing trust following the new positive information. We conducted a series of ANOVAs on ratings of trustworthiness, cognitive trust, affective trust, general trust, and willingness to risk (results are summarised in Table 2). These analyses showed a sustained difference between the Apology and Denial conditions on all variables considered (although the effect on affective trust did not reach statistical significance, the means were in the expected direction). In other words, participants attributed greater trustworthiness and extended greater general trust, cognitive trust, and willingness to risk to the candidate who apologised for her previous trust violation than to the one who denied it (Figure 2).

**Table 2:** Effect of the first impression manipulation (denial vs. apology) on trust ratings following the new positive information.

Dependent variable	ANOVA results		Descriptive statistics by condition: *M* (*SD*)	
	*F*(1, 202)	Cohen’s *d*, 95% CI	Apology	Denial
Trustworthiness	8.69**	0.41 [0.13, 0.69]	5.14 (0.76)	4.81 (0.84)
General trust	18.47***	0.60 [0.32, 0.88]	5.21 (1.10)	4.50 (1.26)
Cognitive trust	13.08***	0.51 [0.22, 0.78]	5.24 (1.02)	4.66 (1.29)
Affective trust	3.72	0.27 [−0.006, 0.54]	4.82 (1.25)	4.49 (1.22)
Willingness to risk	23.48***	0.68 [0.39, 0.96]	5.29 (1.18)	4.41 (1.39)
Aggregate index of trust	8.63**	0.41 [0.13, 0.68]	4.84 (0.68)	4.55 (0.72)

^**^ *p* < 0.01, ^***^ *p* < 0.001.

### Anticipated team dynamics

#### Effect of the first impression manipulation

**Figure 3:**
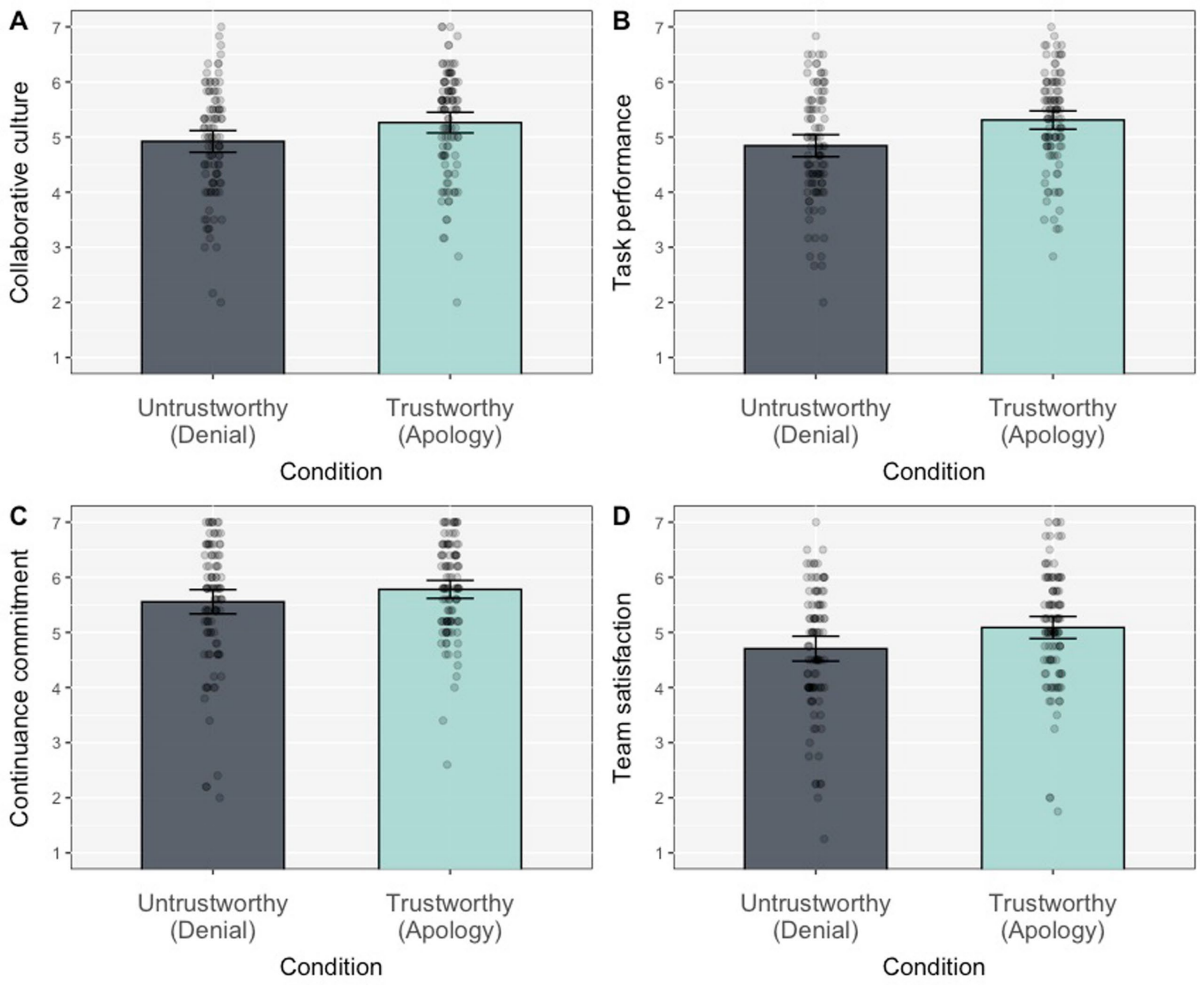
Effect of the first impression manipulation (condition: denial vs. apology) on anticipated team dynamics: collaborative culture **(A)**, anticipated task performance **(B)**, continuance commitment **(C)**, and team satisfaction **(D)**. All differences were significant at *p* < 0.05 except for continuance commitment **(C)** where it was not significant.

We then tested the effect of the first impression manipulation on anticipated team dynamics. A series of ANOVAs tested for this effect on perceptions of collaborative culture (H3a) and team performance (H4a), continuance commitment (H5a), and satisfaction with team functioning (H6a). Results are summarised in Table 3. Consistent with our preregistered hypotheses, participants in the Apology condition reported higher collaborative culture, higher expected team performance, and higher satisfaction with team functioning than those in the Denial condition (Figure 3). Only the effect on continuance commitment did not reach the threshold for significance, although means were going in the expected direction.

**Table 3:** Effect of the first impression manipulation (denial vs. apology) on anticipated team dynamics.

Dependent variable	ANOVA results		Descriptive statistics by condition: *M* (*SD*)	
	*F*(1, 202)	Cohen’s *d*, 95% CI	Apology	Denial
Collaborative culture	6.20*	0.35 [0.07, 0.62]	5.26 (0.96)	4.92 (0.99)
Team performance	12.68***	0.50 [0.22, 0.77]	5.31 (0.86)	4.84 (1.01)
Continuance commitment	2.68	0.23 [−0.04, 0.50]	5.78 (0.84)	5.56 (1.11)
Satisfaction	6.39*	0.35 [0.07, 0.63]	5.09 (1.03)	4.71 (1.14)

^*^ *p* < 0.05, ^***^ *p* < 0.001.

#### Mediation through trust

We finally turned to mediation tests, investigating whether the effect of the first impression manipulation on team dynamics indicators was mediated by increased trust (H3b, H4b, H5b, H6b). We also hypothesised that the effect on satisfaction with team functioning would be serially mediated through trust first and perceived team performance second (H6c). For simplicity, these analyses relied on an aggregate index of trust encompassing trustworthiness, general trust, cognitive trust, affective trust, and willingness to risk (measured after the new positive information was disclosed). Indeed, all measures showed high inter-correlations, and we did not have specific hypotheses about a mediation through one type of trust but not another. We thus deemed it reasonable to work with an overall index.

**Figure 4:**
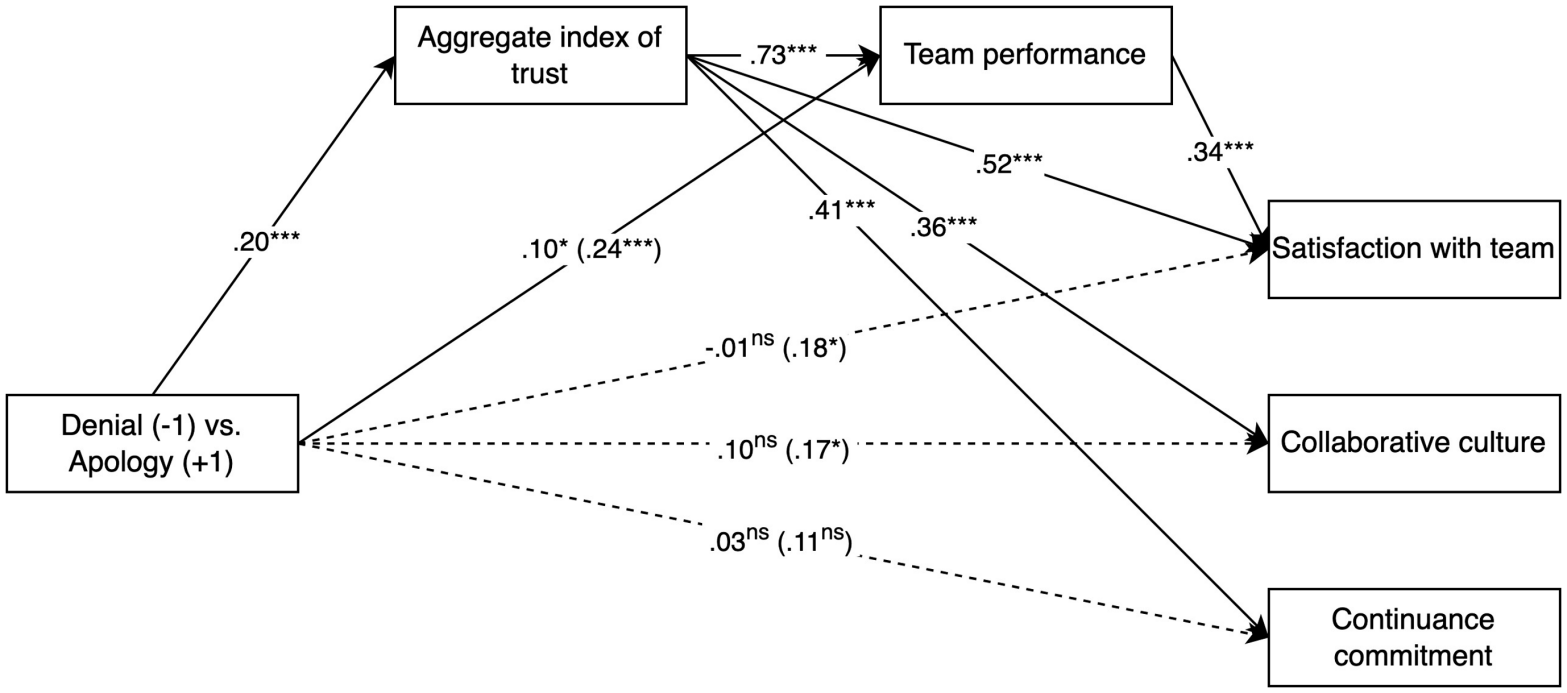
Test of the mediation of the effect of the first impression manipulation (denial vs. apology) on team dynamics indicators through trust. Values reported are standardised coefficients (β). Dashed lines represent non-significant effects. The total effect of first impression manipulation (denial vs. apology) is reported in brackets. * *p* < 0.05, *** *p* < 0.001.

We relied on structural equation modelling (SEM), testing a model which included all team dynamics indicators as outcomes, the first impression manipulation as the independent variable (coded: −1 = Denial, +1 = Apology), and the aggregate index of trust as the mediator. Team dynamics indicators were allowed to covary. We conducted a joint significance test to examine the component paths and then employed a bootstrap resampling method to assess the magnitude of the indirect effect (percentile bootstrap confidence intervals, see Yzerbyt et al., 2018). Results are summarised in Table 4 and the model is depicted visually in Figure 4.

**Table 4:** Test of the mediation of the effect of the first impression manipulation (condition: denial vs. apology) on team dynamics indicators through trust.

Regression model	Estimate	*SE*	*z*-test	*p*-value	95% CI	Stand. β
Trust ~						
Condition	0.288	0.098	2.95	0.003	[0.097, 0.480]	0.202
Collaborative culture ~						
Trust	0.501	0.091	5.49	< 0.001	[0.322, 0.680]	0.361
Condition (direct resid.)	0.197	0.130	1.51	0.13	[−0.058, 0.451]	0.099
Indirect effect	0.144	0.056	-	-	[0.036, 0.253]	0.073
Total effect	0.341	0.136	2.50	0.012	[0.074, 0.608]	0.173
Team performance ~						
Trust	0.987	0.063	15.72	< 0.001	[0.864, 1.11]	0.733
Condition (direct resid.)	0.181	0.089	2.03	0.042	[0.006, 0.357]	0.095
Indirect effect	0.285	0.098	-	-	[0.092, 0.477]	0.148
Total effect	0.466	0.130	3.58	< 0.001	[0.211, 0.721]	0.243
Continuance commitment ~						
Trust	0.569	0.089	6.36	< 0.001	[0.394, 0.744]	0.413
Condition (direct resid.)	0.061	0.127	0.48	0.63	[−0.189, 0.310]	0.031
Indirect effect	0.164	0.061	-	-	[0.044, 0.284]	0.084
Total effect	0.225	0.137	1.65	0.10	[−0.043, 0.493]	0.114
Satisfaction ~						
Team performance	0.387	0.064	6.08	< 0.001	[0.262, 0.511]	0.343
Trust (direct resid.)	0.792	0.089	8.87	< 0.001	[0.617, 0.967]	0.523
Condition (direct resid.)	−0.024	0.091	−0.27	0.79	[−0.203, 0.154]	−0.01
Indirect effect	0.110	0.042	-	-	[0.028, 0.192]	0.051
Total effect	0.384	0.149	2.58	0.010	[0.093, 0.675]	0.178

Fully left justified constructs (~) are dependent variables. 95% confidence intervals are percentile bootstrap confidence intervals.

The analysis provided strong support for our hypotheses. Trust was significantly related to each of the team dynamics indicators and the indirect effect of the first impression manipulation (“condition”) through trust was significant in three out of four cases (i.e., the 95% percentile bootstrap confidence intervals did not include zero). Specifically, trust fully mediated the effect of the manipulation on perceived collaborative culture and partially mediated its effect on subjective team performance. The serial mediation through trust and then team performance also fully mediated the effect of the manipulation on satisfaction with team functioning.

The indirect effect on continuance commitment was technically significant; however, as the total effect of the manipulation on this variable was not, the indirect effect appears to rely on non-shared parts of variance, and we do not interpret it as indicating an actual mediation (Yzerbyt et al., 2018).

## General discussion

Trust plays a vital role in organisational settings. A substantial body of research shows that trust fosters cooperation, productive work relationships, and satisfaction and commitment towards the working environment, as well as greater team performance (Barczak et al., 2010; Costa, 2003; Costa et al., 2018; Erdem et al., 2003; McAllister, 1995). Conversely, trust violations have been found to harm negotiation and bargaining outcomes (Croson et al., 2003; Lewicki and Polin, 2013), decrease cooperation (Bottom et al., 2002; Lount et al., 2008), diminish organisational commitment (Robinson and Rousseau, 1994), lead to negative emotions and desire for revenge (Bies and Tripp, 1996), and in more severe cases, trigger organisational failure (Gillespie and Dietz, 2009).

The present study investigated how the expected (and imagined) arrival of a new coworker can shatter interpersonal trust and consequently team dynamics when the trustworthiness of this person is questionable. Taking advantage of an experimental design, we developed a bogus job interview paradigm to manipulate first impressions through a job candidate’s response to an accusation of past competence-related trust violation (Apology vs. Denial). To investigate how first impressions and belief updating interact, this was followed by new positive information about the job candidate. As outcomes, we measured perceived trustworthiness and willingness to trust the job candidate, as well as anticipated team dynamics, if she were to join the participant’s team.

### First impressions and belief updating

At the theoretical level, the primary goal of the present research was to shed light on the dynamics of trust formation in the work context, specifically testing how first impressions and new information interact to determine interpersonal trust. Consistent with confirmation bias research (e.g., Cvetkovich et al., 2002; Pilditch et al., 2020; Poortinga and Pidgeon, 2004), we expected participants to treat the new positive information in a way that was congruent with their first impression based on the candidate’s behaviour during the interview (i.e., denial or apology). This implies that trust would increase to a greater extent when the first impression was positive. Although the apology response led to a more positive first impression than denial, consistent with our first preregistered hypothesis (H1) and directly replicating past literature (Ferrin et al., 2007; Kim et al., 2004, 2006), the results revealed no confirmation bias in treating the new information (contradicting H2). Rather, they showed an additive effect, as the piece of positive information ameliorated trust to a similar extent in both experimental conditions.

This additive effect appears elsewhere. For example, Chang et al. (2010) found that a trustee’s consistent trustworthy behaviour in a trust game (as the experimental manipulation of ‘new information’) led to high levels of trust, but also that facial features of high vs. low trustworthiness (as the proxy for ‘first impression’) continued to influence trust.

Different explanations may account for these findings. First, it is likely that the timing of receiving the different pieces of information matters. Early work on confirmation bias considered participants’ *pre-existing* beliefs, combined with a new piece of information (Cvetkovich et al., 2002; Poortinga and Pidgeon, 2004; Slovic, 1993). In contrast, in the present study (as in Chang et al., 2010), the information that contributed to the first impression of the candidate and the new information were presented consecutively. It is possible that dynamics of motivated reasoning (Kunda, 1990) and biased processing of new information (Hilbert, 2012) become stronger when the initial beliefs are well consolidated – explaining why we could not identify them in the present research, as trust beliefs may still have been very malleable. It is worth noting, however, that other work has identified a confirmation bias in trust in a stranger when first impressions (i.e., information about the person’s credibility) and new information were both gathered during the short timeframe of the experiment (Pilditch et al., 2020).

More broadly, this speaks to the ongoing debate in the literature as to whether first impressions only matter during the early stages of a relationship (e.g., Kramer, 1999; Lewicki and Bunker, 1996) or whether they have a lasting influence (Ferrin and Dirks, 2003; McKnight et al., 1998). In a recent series of experiments contrasting short-term and long-term effects, Campagna et al. (2022) successfully demonstrated that initial positive information of trustworthiness buffers against negative updating in a direct paradigm. However, this effect disappears if a two-week gap is introduced after the trust violation, and then, only the effect of the violation remains. The notion of timing is also discussed in recent reviews of trust repair, with Sharma et al. (2022) highlighting that the effect of introducing a delay between the trust violation and trust repair efforts remains unclear. In summary, further research is required to improve our understanding of the temporal dynamics of trust belief updating (Korsgaard et al., 2018; van der Werff and Buckley, 2014).

A second explanation might be that different dynamics underlie the updating of a negative first impression in light of new positive information (as in the present study) and that of a positive first impression in light of new negative information. Researchers have suggested that trust is a dominant response tendency, implying that people are generally more prone to trust than distrust (Katzir and Posten, 2023). Consistent findings from an aforementioned study indicated that a first positive impression of someone’s trustworthiness continues to positively affect perceived benevolence and cooperative behaviour even after untrustworthy behaviour: the effect of the first positive impression endures (Campagna et al., 2022). In another study investigating trust in the recommendations of credible vs. non-credible sources, Pilditch et al. (2020) also found that the tendency to believe (false) information from credible sources was stronger than the tendency to reject (true) information from non-credible sources, which may point again to a general motivation to trust. However, other research has yielded opposite findings. In Chang et al. (2010), participants trusted the least the partner who had initially looked trustworthy (positive first impression) but had later betrayed their trust in an economic game (negative new information).

Overall, evidence is mixed regarding trust belief updating, and it appears that the dynamics at play depend on the direction of that updating (from negative to positive and vice versa), as well as on the nature of the information being processed (e.g., facial cues of trustworthiness, reputation, behaviour, etc.). We noted that research on belief updating has remained somewhat disconnected from the trust repair literature (for recent reviews, see, e.g., Kähkönen et al., 2021; Sharma et al., 2022). Although trust repair constitutes a specific case of belief updating (focusing on ways to rebuild positive beliefs after a trust breach), some insights could be better connected to belief updating in general, especially in terms of trust repair strategies and processes (see also Gillespie and Dietz, 2009).

In general, updating seems to be coloured by a broad motivation to trust and therefore to look for cues of trustworthiness even when doubt creeps in. The apparent dominant nature of trust (Katzir and Posten, 2023) may express itself more strongly when the trustee is a person that participants would expect (or imagine) to interact with again in the future. No motivated reasoning is necessary when one plays a trust game against an anonymous partner whom they will never encounter again. However, when the person is for example a new colleague, in reality (van der Werff and Buckley, 2014) or in an alleged scenario (as in the present study), people might be motivated to convince themselves that this new person is better than they might have initially thought and actively look for cues of trustworthiness to correct their initial negative impression.

### A sustained effect of positive first impression

The present study found a positive effect of apology over denial and a constant positive effect of the additional piece of positive information, resulting in a sustained effect of the positive first impression on trust, trustworthiness, and willingness to make oneself vulnerable. The effect was more pronounced for cognitive trust than for affective trust, which is consistent with the view that cognitive trust is more relevant at the early stages of a relationship while affective trust develops later (McAllister, 1995; Tomlinson et al., 2004). Apart from affective trust, all effects were of medium size (0.41 ≤ Cohen’s *d*_s_ ≤ 0.68).

The same pattern of results emerged as we examined team dynamics: here, too, we found a sustained effect of the positive first impression on perceived collaborative culture (H3a), anticipated team performance (H4a), and satisfaction with team functioning (H6a). Only the effect on continuance commitment (H5a) was non-significant. These effects were relatively smaller (0.35 ≤ Cohen’s *d*_s_ ≤ 0.50), which is potentially attributable to the nature of the variables, as they move further away from the core concept of trust and are, rather, consequences of it. Participants’ ratings were also most likely influenced by their perceptions of their own team, beyond the manipulated information about the projected new team member. This is reassuring in a way as the difference in effect sizes suggests the team-related outcomes do not merely reflect attitudes towards the new team member, but rather participants’ integrated view of how the member might affect the team. However, these outcomes remain a projection in a fictional scenario, which is discussed in the Limitations section.

Our mediation analyses further supported the hypothesis that the observed effects on subjective team dynamics were due to a differential level of trust in the new team member (mediation through trust was significant for collaborative culture, team performance, and satisfaction; H3b, H4b, H6b). We also replicated previous findings that the effect on satisfaction is serially mediated through trust first, then team performance (H6c; Costa et al., 2001). Overall, our findings therefore strengthen the idea that interpersonal trust within a team is important for team perceptions.

### Practical implications

At the applied level, we sought to investigate how team perceptions may be challenged by the arrival of a new team member, an event that has been recognised as a potential challenge for teams (Allen and Meyer, 1990; Bauer et al., 2007; Liu et al., 2023) especially when the trustworthiness of this person may be questioned. Our results identified clear effects on team perceptions, highlighting that the anticipated arrival of a new team member can be a challenging time to negotiate. They suggest that it would be essential to provide team members with support, transparent information, and a space to express their concerns, thereby facilitating a smooth transition to the new team composition.

Trust violations are inevitable in the workplace, and at some point, one may need to disclose past trust violations, such as to a new employer. Consistent with previous research, our findings suggest that the best way to handle such disclosure is for one to own their previous mistake, provide an explanation and reassurance, and to apologise for the violation. This holds, at least, as long as the violation is competence-based (Ferrin et al., 2007; Kim et al., 2004, 2006). Importantly, we also showed that subsequent evidence of one’s trustworthiness will continue to ameliorate this first impression. Providing such evidence may be a clever strategy for both the newly hired team member (see also Kim et al., 2013) and team leaders or managers who can reassure team members of their hiring choice and ensure the newcomer is well integrated into the team (Sharma et al., 2022).

## Limitations and future directions

Some limitations of the present work must be acknowledged. We decided to rely on an experimental design to fully control the information given to participants about the trustee and the situation. While this design has clear strengths, allowing us to pinpoint specific causal mechanisms, it is also limited in the amount of information that could be incorporated. As such, the paradigm does not capture the complexity of trust formation in real-world settings where direct communication and experience play a crucial role in trust formation. Future studies that rely on real workplace experience would be a welcome extension to the present findings. Such studies may also take into account other factors from the trustor’s perspective, such as their trust propensity (Rotter, 1971) or past experiences of trust violation in the workplace (Baer et al., 2018; Lalot, 2023), to provide a more thorough examination of the determinants of trust building and updating.

Our study included several measures of trust-related constructs, notably distinguishing affective from cognitive trust and also trust from trustworthiness. However, the measures did not allow for a clear distinction between low trust and clear distrust. This distinction remains a point of contention in the current trust literature with different theoretical perspectives holding either that trust and distrust form a two-pole continuum (with mistrust being the neutral middle-point, Sitkin and Bijlsma-Frankema, 2018; Ullmann-Margalit, 2004), or that trust and distrust are distinct constructs leading to different, specific responses (Bertsou, 2019; Lewicki et al., 1998; Six and Latusek, 2023; see also Fletcher et al., 2024). Future work is needed to better disentangle changes in trust, distrust, and mistrust when it comes to belief updating.

To ensure a sufficient sample size (considering that we were recruiting employees and not a convenience sample of students), we also kept the paradigm simple by comparing only two conditions: Apology vs. Denial. These always related to a competence-based violation, and were always followed by new positive information (consistent with an extensive literature on trust repair). As our results revealed, the dynamics of trust belief updating are complex and significantly influenced by contextual factors. Therefore, it will be important for future studies to extend the present findings in different directions. First, integrity-based violations should be investigated. Previous studies have shown that denial may be more effective than apology in restoring trust (as long as doubt remains regarding the accused’s culpability; Ferrin et al., 2007; Kim et al., 2004, 2006). It would be interesting to investigate how new positive information affects beliefs updating after such a violation. Second, one may want to vary the valence of the new piece of information and consider *both* positive and negative information. This would further our understanding of how and to what extent a positive first impression can buffer against new negative information (e.g., Campagna et al., 2022).

Given the experimental nature of the study, we only considered anticipated team dynamics reflecting the imagined arrival of the new team member. Longitudinal studies, moving back to real-world settings, would be useful for further investigating what happens when a real newcomer joins the team. Such studies could effectively document the longitudinal aspects of team trust dynamics, such as maintenance, strength, or adaptation after an observed trust violation (see, e.g., Campagna et al., 2022; van der Werff and Buckley, 2014). In real-world settings, team members may meet and discuss the trust violation in ways that reinforce negative feelings, ultimately making trust harder to repair (Kähkönen et al., 2021; Kim et al., 2013). Such possibilities need to be explored further.

Finally, it will be crucial for future studies to consider the changing nature of the workplace. Since the COVID-19 pandemic, organisations have been increasingly adopting virtual or hybrid work models (Gilson et al., 2014; Martin et al., 2022). This means that first impressions in the workplace are often formed through digital means nowadays (such as video interviews and asynchronous communication) rather than through in-person interactions, as used to be the only possibility. These channels differ in the richness of the information they convey, as well as its availability, speed of dissemination, and so on. Third-party information can also spread in various ways, from face-to-face gossip at the coffee machine to information shared on a large scale via social media (Bozoyan and Vogt, 2016). These shifts suggest that trust formation and repair processes may be evolving, and future research will need to explore how digital contexts and contemporary team structures influence the dynamics of trust belief updating (see Breuer et al., 2016).

## Conclusion

The present study sheds new theoretical light on the processes of trust belief updating as a function of first impressions and the processing of new information. Our results reveal an additive, rather than multiplicative, effect of new positive information, and highlight that trust belief updating is more complex than sometimes assumed; it does not amount to a mere congruence bias. At the applied level, we highlight how the arrival of a newcomer is a challenging time for a team, even at the anticipation stage. Finally, our findings suggest that trust within teams may evolve, responding to individual perceptions and reinforcement as team members gather more information and insights about one another. These insights contribute to the theoretical foundation of trust dynamics within organisational teams and offer practical implications for enhancing team collaboration and satisfaction.

## Data Availability

The datasets presented in this study can be found in online repositories. The names of the repository/repositories and accession number(s) can be found at: https://osf.io/vyzkx.

## Footnotes

^1^About half the sample (51.5%) originated from the partnering company, while the 48.5% others came from 86 different compagnies (*n* = 1–4 per company). 35.8% had joined their current working team one year ago or less, 31.9% between 1–3 years ago, 14.2% between 3–5 years ago, and 18.1% more than 5 years ago. Forty (19.6%) occupied a management role while 164 (80.4%) were employees.

^2^Similar results were obtained on the indices of trust and trustworthiness considered separately (trust: *F*(1, 202) = 28.57, *p* < 0.001, *d* = 0.75 [0.46, 1.03]; trustworthiness: *F*(1, 202) = 17.88, *p* < 0.001, *d* = 0.59 [0.31, 0.87]).

^3^Considering trust and trustworthiness separately revealed a nonsignificant difference for trustworthiness, *F*(1, 202) = 2.15, *p* = 0.14, *d* = 0.21 [−0.07, 0.48], *M*_Apology_ = 0.23 (*SD* = 0.76), *M*_Denial_ = 0.08 (*SD* = 0.76). A small effect appeared with the single item of trust, *F*(1, 202) = 5.30, *p* = 0.022, *d* = 0.32 [0.04, 0.59] that went in the direction opposed to prediction: trust ratings ameliorated more in the Denial condition (*M* = 0.54, *SD* = 0.94) than in the Apology condition (*M* = 0.26, *SD* = 0.80).
