# Supplementary material for: Of first impressions, shattered trust, and apology: impact on interpersonal trust and team dynamics
Source: Front Psychol. 2025 Aug 7;16:1654463. doi: 10.3389/fpsyg.2025.1654463 (PMC12369420; doi:10.3389/fpsyg.2025.1654463)
Supplement: Supplementary file 1 [file Table_1.docx]

**Of First Impressions, Shattered Trust, and Apologies: Interpersonal Trust and Team Dynamics**

**Electronic Supplementary Materials**

# ESM2. Measurement Model

## Manipulation Checks

A first exploratory factor analysis considered the five items included as manipulation checks. We relied on a multiple criteria approach to determine the number of factors to extract (Ruscio & Roche, 2012). There was a consensus between the comparison data index, parallel analysis, sequential χ2 model tests, and lower bound of the RMSEA 90% CI to recommend extracting two factors. We therefore conducted an EFA with extraction fixed on two factors (maximum likelihood). The solution (explaining 77% of variance) revealed a first factor grouping the three items of trustworthiness and that of general trust (61% variance, all loadings > .48) and a second factor on which the willingness-to-risk item loaded alone (16% variance, loading = .99). We therefore aggregated the four first items in an index of trust/trustworthiness, maintaining willingness to risk as a separate, single-item indicator.

**Table S1.1.** *Rotated Loadings of the EFA on Manipulation Check Items*

| **Item** | **Factor 1** | **Factor 2** |
| --- | --- | --- |
| Integrity | .903 |  |
| Ability | .549 | .342 |
| Benevolence | .634 |  |
| Willingness to risk | .016 | .986 |
| General trust | .487 | .480 |

*Note*. Loadings < .30 are not displayed.

## Trust Ratings

Given the greater number of items used for trust ratings after the extra information about the job candidate had been revealed, we relied on a confirmatory factor analysis instead of EFA. We specified a model where each theoretical construct was composed of several items, except for general trust which consisted in a single item. Further, we specified a higher-order construct of trustworthiness which was composed of the three latent constructs of ability, benevolence, and integrity (see Code S1.1 and Table S1.2); χ^2^ = 581.38, df = 240, χ^2^/df = 2.42, CFI = .912, RMSEA = .084, 90% CI [.075, .092], SRMR = .062.

**Code S1.1.** *Details of the First Model Specified for CFA on Trust Ratings*

cfa.trustT2 <- '

A =~ t2_abil_1 + t2_abil_2 + t2_abil_3 + t2_abil_4

B =~ t2_ben_1 + t2_ben_2 + t2_ben_3 + t2_ben_4 + t2_ben_5

I =~ t2_int_1 + t2_int_2 + t2_int_3 + t2_int_4

trustwo =~ A + B + I

cogtrust =~ t2_cogtru_1 + t2_cogtru_2 + t2_cogtru_3 + t2_cogtru_4

afftrust =~ t2_afftru_1 + t2_afftru_2 + t2_afftru_3

willrisk =~ t2_willrisk_1 + t2_willrisk_2 + t2_willrisk_3

gentrust =~ t2_gentrust

'

This led us to remove four underperforming items (1 ability, 2 benevolence, 1 cognitive trust), χ^2^ = 388.74, df = 158, χ^2^/df = 2.46, CFI = .938, RMSEA = .085, 90% CI [.074, .095], SRMR = .050 – see Code S1.2 and Table S1.3.

**Code S1.2.** *Details of the Second Model Specified for CFA on Trust Ratings*

Rcfa.trustT2 <- '

A =~ t2_abil_1 + t2_abil_2 + t2_abil_3

B =~ t2_ben_1 + t2_ben_3 + t2_ben_5

I =~ t2_int_1 + t2_int_2 + t2_int_3 + t2_int_4

trustwo =~ A + B + I

cogtrust =~ t2_cogtru_1 + t2_cogtru_2 + t2_cogtru_3

afftrust =~ t2_afftru_1 + t2_afftru_2 + t2_afftru_3

willrisk =~ t2_willrisk_1 + t2_willrisk_2 + t2_willrisk_3

gentrust =~ t2_gentrust

'

**Table S1.2** *Results of the First CFA on All Trust Rating Items*

| **Latent variables** | **Estimate** | ***SE*** | ***z*-test** | ***p*-value** | **Standard. *β*** |
| --- | --- | --- | --- | --- | --- |
| A =~ |  |  |  |  |  |
| t2_abil_1 | 1.000 |  |  |  | 0.807 |
| t2_abil_2 | 1.356 | 0.083 | 16.413 | < .001 | 0.956 |
| t2_abil_3 | 1.117 | 0.080 | 13.884 | < .001 | 0.830 |
| *t2_abil_4** | *-0.566* | *0.108* | *-5.259* | *< .001* | *-0.368* |
| B =~ |  |  |  |  |  |
| t2_ben_1 | 1.000 |  |  |  | 0.872 |
| *t2_ben_2** | *-0.139* | *0.117* | *-1.187* | *.235* | *-0.087* |
| t2_ben_3 | 0.931 | 0.062 | 14.896 | < .001 | 0.831 |
| *t2_ben_4** | *-0.562* | *0.117* | *-4.826* | *< .001* | *-0.342* |
| t2_ben_5 | -0.832 | 0.086 | -9.713 | < .001 | -0.620 |
| I =~ |  |  |  |  |  |
| t2_int_1 | 1.000 |  |  |  | 0.857 |
| t2_int_2 | 0.888 | 0.058 | 15.288 | < .001 | 0.830 |
| t2_int_3 | 1.005 | 0.061 | 16.551 | < .001 | 0.880 |
| t2_int_4 | 1.034 | 0.075 | 13.773 | < .001 | 0.792 |
| trustwo =~ |  |  |  |  |  |
| A | 1.000 |  |  |  | 0.846 |
| B | 1.307 | 0.116 | 11.309 | < .001 | 0.947 |
| I | 1.370 | 0.122 | 11.191 | < .001 | 0.948 |
| cogtrust =~ |  |  |  |  |  |
| t2_cogtru_1 | 1.000 |  |  |  | 0.840 |
| t2_cogtru_2 | 1.041 | 0.074 | 13.974 | < .001 | 0.809 |
| t2_cogtru_3 | 1.176 | 0.075 | 15.651 | < .001 | 0.877 |
| *t2_cogtru_4** | *-0.726* | *0.112* | *-6.497* | *< .001* | *-0.450* |
| afftrust =~ |  |  |  |  |  |
| t2_afftru_1 | 1.000 |  |  |  | 0.726 |
| t2_afftru_2 | 1.189 | 0.090 | 13.279 | < .001 | 0.934 |
| t2_afftru_3 | 1.154 | 0.088 | 13.088 | < .001 | 0.927 |
| willrisk =~ |  |  |  |  |  |
| t2_willrisk_1 | 1.000 |  |  |  | 0.805 |
| t2_willrisk_2 | 1.107 | 0.082 | 13.473 | < .001 | 0.816 |
| t2_willrisk_3 | -1.040 | 0.074 | -14.113 | < .001 | -0.859 |
| gentrust =~ |  |  |  |  |  |
| t2_gentrust | 1.000 |  |  |  | 1.000 |

*Note*. Items marked with an asterisk were removed from the final model.

**Table S1.3** *Results of the Second CFA on Trust Rating Items*

| **Latent variables** | **Estimate** | ***SE*** | ***z*-test** | ***p*-value** | **Standard. *β*** |
| --- | --- | --- | --- | --- | --- |
| A =~ |  |  |  |  |  |
| t2_abil_1 | 1.000 |  |  |  | 0.810 |
| t2_abil_2 | 1.346 | 0.082 | 16.398 | < .001 | 0.952 |
| t2_abil_3 | 1.118 | 0.080 | 14.028 | < .001 | 0.834 |
| B =~ |  |  |  |  |  |
| t2_ben_1 | 1.000 |  |  |  | 0.874 |
| t2_ben_3 | 0.925 | 0.062 | 14.882 | < .001 | 0.828 |
| t2_ben_5 | -0.810 | 0.086 | -9.462 | < .001 | -0.605 |
| I =~ |  |  |  |  |  |
| t2_int_1 | 1.000 |  |  |  | 0.858 |
| t2_int_2 | 0.888 | 0.058 | 15.312 | < .001 | 0.831 |
| t2_int_3 | 1.003 | 0.061 | 16.542 | < .001 | 0.879 |
| t2_int_4 | 1.033 | 0.075 | 13.770 | < .001 | 0.792 |
| trustwo =~ |  |  |  |  |  |
| A | 1.000 |  |  |  | 0.850 |
| B | 1.304 | 0.113 | 11.509 | < .001 | 0.951 |
| I | 1.356 | 0.120 | 11.279 | < .001 | 0.946 |
| cogtrust =~ |  |  |  |  |  |
| t2_cogtru_1 | 1.000 |  |  |  | 0.848 |
| t2_cogtru_2 | 1.034 | 0.073 | 14.203 | < .001 | 0.812 |
| t2_cogtru_3 | 1.161 | 0.074 | 15.756 | < .001 | 0.875 |
| t2_cogtru_4 |  |  |  |  |  |
| afftrust =~ |  |  |  |  |  |
| t2_afftru_1 | 1.000 |  |  |  | 0.726 |
| t2_afftru_2 | 1.189 | 0.089 | 13.288 | < .001 | 0.934 |
| t2_afftru_3 | 1.153 | 0.088 | 13.096 | < .001 | 0.927 |
| willrisk =~ |  |  |  |  |  |
| t2_willrisk_1 | 1.000 |  |  |  | 0.805 |
| t2_willrisk_2 | 1.106 | 0.082 | 13.444 | < .001 | 0.815 |
| t2_willrisk_3 | -1.041 | 0.074 | -14.103 | < .001 | -0.860 |
| gentrust =~ |  |  |  |  |  |
| t2_gentrust | 1.000 |  |  |  | 1.000 |
